# Response to: Comment on “Zoonotic and Non-zoonotic Parasites of Wild Rodents in Turkman Sahra, Northeastern Iran”

**Published:** 2018

**Authors:** Monireh GHOLIPOURY, Hamid Reza REZAI, Somayeh NAMROODI, Fatemeh ARAB KHAZAELI

**Affiliations:** 1. Faculty of Fisheries and Environment, Gorgan University of Agricultural Sciences and Natural Resources, Gorgan, Iran; 2. Dept. of Parasitology, Faculty of Veterinary Medicine, University of Tehran, Tehran, Iran

## Dear Editor-in-Chief

It was a great honor to have comments from Professor Sadjjadi on our published article (1). As it was thoroughly stated in the letter, the aim of the study was to identify the endo/ectoparasitic infestation status in the wild captured rodents in Turkman Sahra. So, the authors did not aspire to identify the detected parasite to the species level.

As it was noted correctly, *Angiostrongylus* is considered endemic to Southeast Asia though recent epidemiological studies have proven the expansion of the parasite’s geographical distribution including Egypt, Australia etc. (2–7). Although many studies have attributed this distribution to human activity and the entrance of infected rodents via international transport, natural spread of infected hosts may occur as well (6, 8). Considering the life cycle of *Angiostrongylus*, nearly all rodent *Angiostrongylus* species, inhabit the pulmonary arteries and right ventricle of the heart, the eggs hatch in the lungs and the first-stage larvae filtered out in the lungs and are coughed up, swallowed, and expelled in the feces (10, 11).

It could be expected not to find the adult nematode via loop mediated necropsy, which is mainly performed in epidemiological studies in different regions of Iran, besides most of these studies did not searched lungs for parasitic infestations (11–16).

As long as in our study, no parasitic larvae in feces or blood could be found in the two infested mice, the parasite in the lung smears were considered as the lungworm, *Angiostrongylus*.

The authors deeply acknowledge that further studies are essentially required to identify the true status of the parasite in the region.
